# The Sense of School Belonging and Implementation of a Prevention Program: Toward Healthier Interpersonal Relationships Among Early Adolescents

**DOI:** 10.1007/s10560-013-0305-5

**Published:** 2013-04-19

**Authors:** Marie Drolet, Isabelle Arcand, Daphne Ducharme, Raymond Leblanc

**Affiliations:** 1 University of Ottawa, Ottawa, ON Canada; 2 University of Ottawa, 120 University Street, Room 12022, Ottawa, ON K1N 6N5 Canada; 3 Ottawa, ON Canada

**Keywords:** Early adolescents, Healthy peer relations, Promotion of social skills, Prevention of alcohol and drug use, Qualitative methodology, Evidence-based practice

## Abstract

The purpose of this qualitative study is to pave the way for the establishment of healthy interpersonal relations by facilitating an understanding of the impacts of *Lions Quest Skills for Adolescence* as perceived by adolescents and teachers who took part in it. *Lions Quest* has become recognized as an evidence-based program for preventing alcohol and drug use through the development of social skills and the promotion of meaningful engagement in the school community (Lions Clubs International, Overview of Skills for Adolescence 2013). Semi-structured interviews were conducted with 7th and 8th grade Francophone and Anglophone adolescents from three schools in Eastern Ontario who had participated in *Lions Quest*. Deductive and inductive analysis of interview transcripts clearly underscored that the positive perceptions of those early adolescents on the quality of their relationships with friends outweigh the negative impression that can be created by peer pressures at this age. It is through such a filter that these adolescents came to appreciate the impact of *Lions Quest.* Their need to be part of a circle of friends also comes to the fore as a crucial component of a sense of school belonging (Faircloth and Hamm (2005) J Youth Adolesc 34:293–309), highlighting the need to incorporate more of this form of positive social norm into interventions and prevention geared toward early adolescents. The data also underline the complementary importance attributed to having positive relationships with supportive adults.

Social workers must continually re-examine their roles and tasks based on a dual responsibility that consists of making decisions on solid findings, and evaluating the effects of their actions (Timberlake, Zajicek-Farber and Anlauf Sabatino 2008). From an ethical perspective, the client deserves to receive the best available services, particularly with respect to evidence-based interventions. Fraser et al. (2011) challenge social workers to balance the introduction of evidence-based programs with possible adaptations applicable within various contexts. As for school social work, a meta-analysis of practices underscores its positive impact on the social, behavioral, emotional and academic development of children and adolescents (Franklin, Kim and Tripodi 2009). These authors suggest that interventions should focus more on elements with immediate impacts on young adolescents’ well-being, such as the sense of school belonging.

With regard to adolescents, Fleming et al. (2010) advance that a detachment from school and academic performance, increased exposure to negative peer pressures, and deterioration in their relationships with parents are major risk factors during this phase of development. They thus propose that preventive efforts be focused on these indicators. From this perspective, an Eastern Ontario social services agency initiated the evidence-based program *Lions Quest Skills for Adolescence* (Eisen et al. 2002) in collaboration with the French and English school boards of that bilingual region. These agencies decided to focus, for the most part, on Grade 7 students. The overall goal of this program is to prevent, or at least delay the use of alcohol and drugs by helping adolescents develop and build their social skills (Lions Clubs International 2013). Given that school-based social work subscribes to an ecosystem analysis (Kelly, Frey and Anderson-Butcher 2010; Drolet and Dubois 2008), this article will report on a qualitative investigation giving a voice to various actors from three Eastern Ontario schools where *Lions Quest* was offered. These individuals participated in the program’s implementation from a range of perspectives, hence they will offer diverse points of view.

In a school setting, social workers often collaborate with people from other disciplines, provide support to those who intervene with youth as part of their day-to-day work, or serve these same persons as key sources of information regarding social problems (Lynn, McKay and Atkins 2003; Viggiani, William and Bailey-Dempsey 2002). Using these roles as points of reference, this study will be guided by one research question and two secondary questions:

- How do the various actors involved in *Lions Quest* perceive the program and its implementation?
  - How did the 12-to 14-year-old teens perceive the sense of school belonging?
  - How do these sentiments contribute to *Lions Quest* objectives of risk prevention and promoting social skills?

To answer these research questions, we will first provide a summary of leading research in social prevention (most written from a social work perspective), risk and protective factors, and the sense of school belonging. We will describe the methodology by which we investigated the perspective of concerned actors. We will offer key findings showcasing the views of adolescents who participated in *Lions Quest*, and of the teachers who oversaw its application. We will give various perspectives on the feeling of belonging at school viewed as a protective factor for young people entering adolescence. We will conclude by proposing avenues and practices to promote in terms of school social work and with young adolescents.

## Social Prevention: Advances in Recent Decades

In the field of social prevention geared toward adolescent problem behaviors, research has seen numerous advances in recent decades (Jenson 2010). More precisely, preventive programs drawing upon fear to sensitize teens to the consequences of their actions (such as alcohol and substance abuse) have proven to be much less effective than expected (Hawkins 2006). Programs that focused solely on extracurricular activities and on contact with positive peers and/or trusted adults to counteract the negative influence of certain friends, also demonstrated limited effects (Jenson 2010).

On the other hand, programs that proved to be more successful sought to help adolescents to acquire social skills, make responsible decisions, develop higher self-esteem and resist negative peer pressures (Skiba et al. 2004; Gottfredson and Wilson 2003). With regard to interventions designed for school settings, a meta-analysis of programs addressing peer influences and prevention of substance use demonstrated that interventions rooted in participation and the acquisition of social skills reached their objectives more readily than did conventional methods.

The research of recent years, including literature in social work, underscores the importance of programs that call for an ecosystem analysis and a strengthening of protective factors (Hawkins 2006; Hill 2008; Jenson 2010). Preventive services should promote solid ties between young people and their schools, and their families, in order to provide opportunities for them to become involved in a favorable manner, develop various skills, and benefit from encouraging positive reinforcement from within their social sphere when their actions are praiseworthy (Hawkins 2006). The school setting is recognized as an excellent environment in which many preventive programs are in effect (Shears, Edwards and Stanley 2006; Weisz et al. 2005) and becomes a center of life experience wherein diverse protective factors can support adolescents’ positive development (Brooks 2006).

## Risk and Protective Factors in Terms of Alcohol and Drug Use

Given that an ecosystem analysis focuses on an individual within his or her diverse surrounding systems (Payne 2005; Moen 1995), Wright, Bobashev and Folsom (2007) indicate that even if the community in which young people live has an influence on whether they consume or not, individual factors such as self-confidence or self-assertion bear considerable weight in making choices. For example, Wills et al. (2011) submit that adolescents with weak self-control are generally more inclined to consume.

From a family angle, adolescents who receive little parental attention or supervision or who have a parent who abuses substances (Catalano and Hawkins 1996) are inclined to consume as well. With regard to the youngest adolescents, Sieving, Perry, and Williams (2000) affirm that the influence exerted by their social groups to become users is reliant upon the tendency of young teens to choose friends whose habits are similar to their own. Equally, adolescents experiencing personal problems will affiliate with others in similar situations (Catalano et al. 2004), selecting them from among a group of peers. Moreover, the desire to belong to a group can influence teenagers’ conduct even before they actually join one; they will already have taken on the behaviors of the peer group in order to ensure acceptance in it (Brown and Larson 2009). Henry, Kobus and Schoeny (2011) indicate that teens have an opinion as to whether their friends consume alcohol or drugs in terms of their own consumption.

Below-average academic achievement can play a role in the adoption of at-risk behavior (Kostelecky 2005) in the same manner as can negative experiences, non-participation in activities, and a general feeling of disengagement with regard to the school (Case 2007). On the other hand, the sense of school belonging evolves into an important protective factor against substance use, especially in relation to illicit drugs (Ford 2009; McNeely, Nonnemaker and Blum 2002). This applies especially to girls in their early teens, students in Grades 7 and 8 (Oeslner, Lippold and Greenberg 2011), and adolescents who have access to limited extracurricular activities outside those offered by schools (Shears et al. 2006). Since the sense of school belonging can have a protective impact—and the age group embraced by the preventive program *Lions Quest*—we will direct our attention toward them.

## Sense of School Belonging

According Faircloth and Hamm (2005), the sense of belonging that young persons have in terms of their school comprises: (1) a network of positive friends through which the young adolescent feels recognized; (2) a positive tie with teachers or other adults through which the adolescent feels appreciated, supported, and assured of help in difficult times; and (3) participation in extracurricular, cultural or sports activities, among others. Involvement in various extracurricular activities has been reported to result in a heightened sense of school connectedness (Dotterer, McHale and Crouter 2007; Durlak, Weissberg and Pachan 2010). A respectful atmosphere and a policy of authoritarian discipline are also associated with a stronger sense of attachment to school (McNeely et al. 2002). Clear, shared expectations, norms that speak of effort and accomplishment, constructive values and positive reinforcements all pave the way for the development of relations of trust within one’s environment and offset the negative influences of other peers encountered at school (Lindstrom 2008).

McNeely and Falci (2004) go a step further and advance how important it is to consider the actual type of ties a young adolescent forges at school. They make a distinction between conventional attachment (i.e., with teachers or other students who demonstrate prosocial behavior) and unconventional (with students who bring a non-conformist element into prosocial norms). Whereas conventional attachment promotes healthy comportment, the unconventional form tends to open up opportunities for adopting at-risk behaviors that may jeopardize the forming of ties. More specifically, the support from teachers or other adults that encourages engagement within the school setting serves as a block against initiation into at-risk behavior. Yet such support has little bearing, if any at all, when offered after young teens have already adopted this behavior. On the other hand, if teens make an effort to commit themselves to relationships with their teachers or other adults, those ties might help them to reduce at-risk behaviors (McNeely and Falci 2004). But students who misbehave, obtain low marks, or have friends whose actions do not fall into acceptable social norms, have fewer chances to benefit from that rewarding attachment to school and positive adults that would lead to positive development (Oeslner et al. 2011). Considering those facts, we will adopt Faircloth and Hamm’s (2005) definition as the framework for this article since it highlights the importance of a network of positive friends.

## Program: *Lions Quest Skills for Adolescence*

*Lions Quest* is a multidimensional educational program, developed in the United States, translated into 31 languages and offered in 65 countries. It is the third most important program in the United States directed toward preventing alcohol and drug abuse (Eisen et al. 2002) and among the evidence-based programs recognized by the Center for Substance Abuse Prevention (www.samhsa.gov).

*Lions Quest* seeks to prevent or delay adolescents’ alcohol and drug use by helping them to develop a constructive commitment to their family, school, and community through the development of social skills and competencies whereby they: (1) develop a capacity for self-assertion and for resisting negative peer pressures; (2) acquire a sense of responsibility; (3) develop conflict resolution skills; and (4) learn how to intervene with peers who start to become substance users. Through a series of 30 one-hour sessions, participants meet in classrooms during regular hours, typically during health or physical education periods. They are conducted by the teacher of the class involved, with occasional support offered by a social worker from the social services agency that initiated the project. In Eastern Ontario, the program brought together students primarily from Grade 7, with several from Grade 8, all between 12 and 14 years of age. This age group was the focus of evidence-based research conducted by Eisen et al. (2002) and identified by a meta-analysis of school-based substance abuse prevention programs (Gottfredson and Wilson 2003) as being the most effective given the increase in peer pressures that accompanies the dawn of adolescence.

Finally, our review of the literature highlights that no qualitative study has yet addressed the views or perspectives of adolescents aged 12–14 regarding the prevention of alcohol and drug use and associated issues that affect their daily lives. In this vein, the present article will explore the impact of *Lions Quest* on students from grades 7 and 8 from the points of view of diverse participants, including the young teens themselves and their teachers. We will follow a parallel avenue by examining whether the young participants believe they experience a sense of belonging to their school, how this sentiment is characterized, and what role these elements may play in reaching the program objectives.

## Methodology

We designed a qualitative investigation to understand how the actors involved in *Lions Quest* perceive the program and its implementation. Semi-structured qualitative interviews were conducted with adolescents who participated in the program as well as the teachers who oversaw its implication. In subsequent pages we further describe the participants, the procedures that guided this study and its limits.

### Participants

First, 26 students aged 12–14 years participated in the study. They attended three schools in Eastern Ontario. These young adolescents formed a voluntary sample, which represents a quota sampling as defined by Mayer and Deslauriers (2000). In order to allow for a diversity of opinions and comparison among three sub-groups of young individuals, we interviewed eight Grade 7 Francophone students in 2009–2010 who had participated during that same year; nine Grade 8 Francophone students in 2009–2010 who had participated in *Lions Quest* in 2008–2009; nine Grade 8 and Grade 9 Anglophone students in 2009–2010 who had participated in the program in 2008–2009. These young teens came from two linguistic communities; the length of time between their participation in the program and the interviews also varies.

The demographic data applicable to the 26 students indicated that, in general terms, they came from two-parent families with both parents in paid employment. The levels of education of the adults ranged from high school to a master’s degree. All were residing in an equal proportion of countryside, suburbs, village or city. All students who submitted a demographic survey were born in Canada and self-identified as either Francophone, bilingual, Canadian or Caucasian. This sampling is typical of the population of the bilingual region of Eastern Ontario.

In addition, five teachers who overviewed the implementation of the *Lions Quest* program participated in the study. They taught in the three different schools. Four of them were employed in Francophone schools and one in an Anglophone school.

### Procedures

The data were collected from January to July 2010. Semi-structured interviews were conducted with students and with teachers. They lasted 45–60 minutes and were recorded for subsequent analysis. They were transcribed and the verbatim statements were imported in the N-Vivo 8 program for coding into themes and categories (Huberman and Miles 1991; Paillé and Mucchielli 2008). The creation of the grid of categories was based on the inter-judge method whereby a portion of the results were read independently by three co-researchers and three graduate students from diverse disciplines. The grid was subsequently agreed upon by consensus between researchers and assistants. Three graduate students coded the transcripts; one focused on the teacher interviews, another worked on the verbatim statements of the Francophone adolescents, and the third concentrated on the perceptions expressed by the Anglophone students. The method of interpretation followed that prescribed by Huberman and Miles (1991): the results of each school were subjected to deductive and inductive analysis in order to conduct an intercase comparison.

### Limits

As with other qualitative research studies, although the saturation level of the data, the uniformity of the results and a diversity of participants were reached, theoretical generalization must be approached with caution (Pires 1997) in view of the small number of participants in this study. However, the insights provided in the interviews, the consensus among the two subgroups (i. e., teens and teachers), and the concordance with the major articles on the topic strengthened the validity of this study (Laperrière, 1997). Nevertheless, it remains difficult to predict with any accuracy the dynamic forces prevailing in other linguistic communities and the long-term effect of the program. Moreover, keeping in mind some leading research on adolescent development, the results of these interviews with the 12- and 14-year-olds are sometimes general in nature as the participants are less inclined to make cause-and-effect links or explore them in depth since concrete thought is still in a development phase and in gradual progress toward abstract thought; a capacity for introspection will blossom fully at a later stage of maturation (Cloutier and Drapeau 2008). Even with those limits, some consensus clearly emerges between the points of view of the diverse participants.

## Findings

The following section presents the findings of our analysis. Specifically, these results are presented in two subsections. The first concerns the tools that favor the development of young adolescents, which are presented from the perspective of the teachers and from the perspective of the adolescents themselves. Then, the second subsection addresses friends as a central preoccupation for young adolescents, the ties between friendships and school connectedness, and the filter through which the adolescents appreciated the impact of *Lions Quest*. Numerous quotations are presented in the findings sections as they offer support to the analysis that took place. The volume of quotations is also crucial to this qualitative study as it allows readers to develop their own interpretations of the story (Stake 1995, 2005).

### Tools Favoring the Development of Young Adolescents: Insights from Teachers

Since social workers often work in school settings in a supportive way with teachers who interact daily with teens, it is important from the outset to highlight the perspectives of these collaborators so our interventions will be as relevant as possible. We begin this results section by examining the objectives of *Lions Quest* as perceived by the five teachers who oversaw the program in the three different schools. Following the *Lions Quest* exercise, the teachers’ observations underscored improvements in the adolescents’ self-confidence, their capacity for self-assertion, and their interpersonal relationships. On this last point, the teachers’ observations mirror the link between such ties and a sense of school belonging (Faircloth and Hamm 2005).

#### Self-Confidence

This trait emerges as a dominant theme in the interviews held with school staff. The five teachers related—often and with enthusiasm—how important it is that young adolescents participating in *Lions Quest* experience an increase in the self-confidence they have in their own abilities. As an example, one teacher outlined that he had placed considerable emphasis on raising and reinforcing confidence in oneself and one’s talents in his daily classes, an indication that the program teaching model was particularly stimulating and complementary to his curriculum. *Sandra (pseudonym) was insecure. She often wanted to change schools. She had problems with other girls and got into arguments. She was turning in on herself. She decided to take the situation in hand. How did the program help her through these troubles? Certainly in the area of self*-*confidence, I was able to offer other tools, and this helped her to make appropriate decisions.* (Teacher 1)

#### Capacity for Self-Assertion

The interviews with teachers confirmed that *Lions Quest* helped the young adolescents to better assert themselves and defend their opinions. One teacher cited the case of a tall, strong boy who had been intimidating other students and, thanks to the program, was able to gain a healthy form of self-respect and to express himself in a more sociable fashion. The same teacher spoke as well of another boy who had been the scapegoat of his group but learned to speak up for himself. *He was being picked on, all the time. Now it’s not like that any more because he can defend himself. He does it verbally, by saying, ‘Stop it, I don’t like this!’* (Teacher 1) According to the teachers, *Lions Quest* played a pivotal role in bringing about such changes. One said, *It takes a lot of courage for some of them to take a stand outside of their group. (…) The discussion groups we conducted [through the Lions Quest program] gave them the confidence to speak up for their own beliefs, and they felt like, ‘Hey, this is how I feel, and I may be for or against something, but I’ve got my reasons and it’s OK if they’re different from others.’ I think the students developed their skills considerably.* (Teacher 3)

#### Interpersonal Relationships

The teachers also noticed a difference in relationships among the students under their daily supervision and offered several examples. One spoke of an aggressive boy who learned to verbalize feelings instead of making threatening gestures when angry. *He had a serious behavioral problem. While we were conducting the ‘Lions Quest’ program, there was a moment when everything clicked into place, and he changed. (…) He gives his all when he discusses something, by expressing himself with words instead of acting up when he’s mad.* (Teacher 2) Another indicated that not only did the program help the teens to develop citizenship values through participating in voluntary activities; it became a drawing card through the interest it generated in civic spirit.

Overall, the teaching staff provided the tools with which the young adolescents acquired greater confidence in their abilities and became more adept at expressing their viewpoints in the presence of peers. Knowing their students so well thanks to daily contact, the teachers were able to integrate the program content into classroom routine in the form of group dynamic and, at times, on a one-to-one basis with individual teens. According to the teachers, this method gave them a greater opportunity to apply *Lions Quest* to issues that fall within the daily reality of 12- to 14-year-olds, to realize immediate results, and to see improvements in peer relationships among the students—this last point being a constant concern of young persons of that age group (Brown and Larson 2009). The teachers from all three schools conducted the program in keeping with the analysis of Fraser et al. (2011), who encourage social workers to adapt their actions to the social context of the setting in which they intervene.

### Tools Favoring Positive Relationships with Peers: Insight from Adolescents

Since social workers also intervene regularly with young adolescents in school settings and elsewhere, and few qualitative studies deal with the reflections of 12- to 14-year-olds on day-to-day issues, we will present a detailed account of the results of the interviews with 26 students from Grades 7 and 8 coming from the three participating schools. As will be indicated in the following pages, their perceptions indicated insight into their daily experiences following their participation in *Lions Quest*. Their comments suggested that the program primarily touched upon their interpersonal relationships and peer group activities, both of which are linked to a sense of school belonging. This frame of reference readily became the filter by which the adolescents appreciated the impact of *Lions Quest*. It also had an impact upon the students’ self-confidence, ability to assert themselves, and conflict resolution skills.

#### Interpersonal Relationships

The relational dimension, notably ties among classmates and friends, was clearly placed center stage through the statements of the adolescents we met. They recounted that within the scope of *Lions Quest*, they participated in numerous activities in order to become better acquainted with peers, to socialize with them in a deferential manner, to be less timid and more caring, and to accept and respect others. The students considered that the program activities had the aim of encouraging them to collaborate in a productive fashion and create a team spirit. With the group sessions, these emerging teenagers were able to sense solidarity within a structured, more respectful, amicable and inclusive environment. Some of them stated that they truly benefited from the ties they forged with other adolescents and their effort to improve their relationships in general. *I’d never be that social in a classroom because, like I’m shy and I don’t talk to anybody. (…) But you know, by the end [of Lions Quest program], everybody was beginning to say: ‘You know what, she really is fun to be with, let’s go and hang out with her’.* (Teen 4, Grade 7)

#### Group Solidarity

It appears that the program activities had a favorable influence on cohesion within groups, a theme readily identifiable in the students’ comments. *Ever since ‘Lions Quest,’ after those activities you’d have to say there’s less bullying at school.* (Teen 18, Grade 8) Although only a minority of students addressed the topic of atmosphere at school, these short yet thought-provoking comments leave the impression that the program indeed had an influence on the daily lives of many students and on the social climate within groups. *We’re all close in our class, the whole 8th grade, we’re all one big family. (…) We don’t judge anybody (…) we’re always there for each other. (…) Someone’s accepted for who they are.* (Teen 6, Grade 8)

#### Self-Confidence

The theme of confidence in oneself appears frequently from an analysis of the interviews with adolescents, mirroring much of what was revealed in the teacher interviews. A range of activities guided them toward recognizing their strengths and talents and feeling at ease about revealing these attributes to others. About his general impressions of the program, a 13-year-old boy answered without hesitation: *We found our class fun because the teacher was organizing lots of activities and we learned by doing fun things. Now we’re more confident in ourselves.* (Teen 5, Grade 7)

#### Capacity for Self-Assertion

According to the adolescents interviewed, growth in self-confidence goes hand in hand with an increase in self-assertion. *Interviewer: ‘What is it that makes you have more respect now for your own opinions?’* The 13-year-old student responded: *Probably because I’m more sure of myself. (…) And I know I have a good idea about what I’m doing.* (Teen 6, Grade 7) These new capabilities inspired the students to surpass themselves. *I was starting to flunk math and French. (…) I wanted to get some help even if maybe they’d laugh at me, so I took the risk anyway.* (Teen 17, Grade 8) It became apparent that the adolescents drew much from *Lion Quest* in terms of life lessons given that it presented them with strategies for better self-expression while respecting others at the same time. As for process, although the manner whereby the strategies were applied in some cases did not achieve the desired level of maturity, they nonetheless generated a higher degree of self-awareness. *I usually don’t say it in the well*-*behaved manner that everybody says you should. I usually use a loud voice. We used to have all these programs where it’s, like, telling you how to solve these conflicts and it was always, take time to calm down and then express it to the person calmly.* (Teen 13, Grade 8)

#### Conflict Resolution

The perceptions of the students indicated they also developed certain skills in conflict resolution, their comments indicating that such ability is often tied in with relationships with peers. They gave numerous examples of previous conflicts they had experienced with classmates, including disputes between friends and incidents of bullying. They related anecdotes about how they learned to calm down and better resolve discord, seek the advice of an adult, reach a compromise through discussion, as well as apologize. *(…) I try to solve the problem, then find out what I’m doing that he doesn’t like, because I don’t wanna lose my friend.* (Teen 2, Grade 8) Indeed, the influence of *Lions Quest* in the area of conflict resolution was moderated according to the individual teens being interviewed. Some said they would react in the same fashion if a similar conflict situation presented itself, whereas others attested that *Lions Quest* played a role in improving their conflict resolution skills. *I wasn’t thinking about what I was saying, so just about anything came out, but now I know how others feel if I let loose like that. I’ve learned to be nice. I’ve decided I should think before I speak.* (Teen 6, Grade 7)

Primarily what the students from all three schools retained from the program were the elements calling for reflection upon their relationships with friends. In this angle, their perspectives follow the same direction as those of their teachers. It has become common that the peer group occupies such a predominant place in adolescent life that, in the case of 12- to 14-year-olds, it surpasses that of parents, from whom the offspring strive to distance themselves in order to shape their own identity (Brown and Larson 2009; Claes 2003). It appears that the students did not go so far as to reflect at length on how to resist negative peer pressures, although this is one of the goals of the program. Rather, they grasped the tools that assisted them in improving interpersonal relationships, defining their roles more effectively and establishing prosocial and positive norms within their social groups—in other words, maintaining and developing positive social assets.

## The Center of Preoccupation of Young Teenagers: Friends

As a follow-up to the analysis of the impact of *Lions Quest* on young adolescents, we now address their sense of belonging at school and whether this sentiment contributes to the attainment of the program objectives. Right from the start, it was interesting to note that a 13-year-old immediately focused on ties with peers and school staff, as well as school-based activities, as lying at the root of his attachment with the school environment. To the question, *Do you feel an attachment to your school?* he replied, *Yeah, because like I have lots of friends there, that’s what it’s all about! There’s some good teachers too. I really like it there: we do lots of activities, and I’ve made so many new friends with some people I met there.* (Teen 2, Grade 7) These statements clearly fall in line with the research of Faircloth and Hamm (2005) on the sense of school belonging. The other students in the study conveyed views that were, in general, much like those of that 13-year-old boy. In this regard, the following section and the subsequent discussion will present points of view the young adolescents held concerning this crucial component of a sense of attachment to school (Faircloth and Hamm 2005): their relationships with peers. School-based activities, such as those of *Lions Quest,* become the signpost marking the path toward the creation and maintenance of positive relationships with friends, and then adults.

### Positive Relationships with Peers

Analysis of the adolescents’ comments brings to light that relationships with their peers are closely linked to a sense of well-being in their environment. When asked if they feel at home and connected to their school, they invariably answered within the context of their friendships. From the outset, this number one preoccupation with friends is analogous to the influence that *Lions Quest* exerted, one in which the teachers detected a development. This concordance leads us to build upon that theme in order that the friendship-based component of school connection may contribute even more to the goals of the intervention and the promotion of social skills.

The teens discussed during the interviews that the frequency of social contacts was an important aspect of the stability of their friendships: seeing their closest friends often at school, during extracurricular activities, or outside the school altogether. These adolescents insisted on the qualities they considered to be important in their peers, specifying repeatedly they sought friends who were of respectful character. As an example, one 14-year-old girl stated, *I hang out with nice people. I don’t like being with people who are obnoxious. Don’t like them at all, it’s not nice and I can’t understand why anybody would even want to hang out with them.* (Teen 2, Grade 8)

In the same vein, those adolescents appreciate being able to feel at ease in their immediate environment, thereby benefiting from a freedom from intimidation. Many of them indicated that they and their peers accept and respect differences among themselves, and are thus able to spend time together in their group dynamic in a spirit of mutual understanding. To quote one 14-year-old boy, *It’s cool because the whole gang’s like me, and I’m the same as them. We like everybody a lot, we have fun together, we’re all smart the same way I am and we’re really close. We’re a family.* (Teen 18, Grade 8)

Apart from that well-being, the young adolescents place considerable value upon trust in relationships. It is important to be able to open up to their friends and have their privacy respected. Many affirmed that they share secrets with trusted friends and in return are taken into similar confidence on a one-for-one basis*. I can tell him something and he won’t turn around and blab it all around. And he can tell me stuff too, like give me his opinions and I tell him mine.* (Teen 1, Grade 8) A large number of teens reinforced the viewpoint that a circle of friends provides mutual support. They stressed the fact that young people need to provide a positive social network and underscored the importance of looking out for each other’s welfare. They cited many examples whereby a friend who was living through a difficult situation or experiencing negative emotions received support from the others whose presence brought comfort, distraction, or much-needed laughter. These findings flesh out the conviction that such mutual assistance and acceptance among peers is the foundation of a positive social network.

In essence, respect, trust in each other, and mutual support are the fine points that young adolescents hold in high regard among their friends. When these traits bind a group of friends, they lead to shared ties through participation in activities. In this regard, Brown and Larson (2009) specify that a group of adolescent peers defines itself as an association of people who view each other as equals and who share a common lifestyle. Moreover, Buchanan and Bowen (2008) maintain that social support offered among friends is considered to be an important source of adolescent well-being. As will be discussed below, these elements could well be integrated into prevention programs (including *Lions Quest*), complement social work interventions with young adolescents, and provide the guideposts leading to social norms and a more profound understanding of their daily life experiences.

## Discussion: Indicators for Social Work Practice

This article set out to facilitate an understanding of the perceptions of various actors on the impacts of *Lions Quest* on 12- to 14-year-olds, as well as to determine whether any aspects of their sense of school belonging contributed to the achievement of the actual program objectives. The verbatim indicate that, beyond the expected capacity to resist peer pressures as originally set forth by the program, the development of social skills and engagement to their school community that the young adolescents carried forth from this exercise encouraged them to think about the quality of their relationships with friends, and even more so, to improve them. Interestingly, they listened more attentively when adults addressed this issue that they considered to be crucial in the context of their own frame of reference and their day-to-day experiences. We therefore come face to face with their need to be part of a milieu, of a niche finding and of a circle of friends, the latter having moved to the forefront as a crucial component of a sense of school belonging (Faircloth and Hamm 2005).

For adolescents to sense that they truly belong within a certain group, they must: (1) have a tangible affiliation with members of that group; (2) benefit from a feeling of self-esteem stemming from involvement with the group; and (3) believe that they are appreciated by the other members and take pride therein (Newman, Lohman and Newman 2007). Brown and Larson (2009) advanced that a peer group confers a social status to an adolescent, a level of popularity, and influence in the eyes of others. Indeed those with the most developed social skills have a greater chance of being part of a peer group held in the highest regard by other young adolescents (Brown and Larson 2009). The desire to belong to a select group can also influence the behavior of young adolescents even before they are welcomed into the inner circle, in order to guarantee acceptance. Accordingly, the findings put us at the heart of the combined processes of selection (“people like me”) and socialization (interinfluence) (Brown and Larson 2009)—factors that many assert as peer pressures and the participants qualify as group belonging.

Given the young participants’ need for a deep sense of belonging (or in other words, to establish meaningful ties with their friends), the young adolescents in this study entered into a phase of reflection vis-à-vis *Lions Quest* (Ryan and Deci 2000). Their desire to belong led to a spirit of greater openness, such motivation being rooted in three psychological needs according to the self-determination theory: relatedness, autonomy and competence (Ryan and Deci 2000). Moreover, by relying on a profound need for competence, they became more disposed towards self-development and achieving higher social skills. Indeed, their hopes of being seen as competent and valued within their peer group served to reinforce the above-mentioned process. It is equally important that adolescents present at least a basic level of autonomy in order to become motivated. They must therefore make enlightened choices and decisions in a responsible fashion, as advocated by the program. As well, if adolescents are to become motivated to forge solid bonds with positive people (prosocial peers or adults), the stability of those relationships depends upon: (1) opportunities that the adolescents perceive for engaging in stimulating activities and forming enriching ties; (2) the actual degree of involvement required; (3) the skills called for; and (4) especially the positive reinforcement that emerges from within the framework of the relationships and activities (Catalano and Hawkins 1996). When they invest in a friendship, young people will in fact take into account the costs and benefits of their choices, as well as the consequences thereof in terms of the persons who are closest to them and whose opinions they respect the most, including their friends. Throughout the analysis of the interviews, the perceptions of the young adolescents clearly overcame the negative impression that peers can generate at this age. They shed light on the importance of having friends and the support they offer one another, offering adults a way to reach them and intervene in their frame of reference.

Notwithstanding all these considerations, it should be specified that even when adolescents experience well-being thanks to supportive friends, such benefits can be compromised if the support offered by adults is weak, even more so if the younger ones are passing through a time of personal difficulty (Buchanan and Bowen 2008). In the same vein, the adolescents stated clearly during the interviews that the adult always remains a key player and it is important to have a positive relationship with an adult, a teacher, or a social worker. This is the other pivotal component in their sense of school belonging as defined by Faircloth and Hamm (2005).

To conclude, these young adolescents had begun to feel that, through *Lions Quest*, they were becoming a valued part of a larger “whole” with talent, potential, and ventures to pursue. In their comments, they enshrined what researchers in social prevention advance: get involved in a positive fashion at least at school; develop skills and solid ties; and benefit from encouraging positive reinforcement from various actions within one’s environment, from positive friends and prosocial adults (Brooks 2006; Hawkins 2006). The perceptions of the teens that largely touched upon the sense of school belonging underscored the need to incorporate this form of well-being and positive social norms into interventions geared toward the prevention of at-risk behaviors (substance use or other difficulties), all within the context of early adolescence. Social workers, teachers, school professionals, adult mentors, parents and researchers all share the goal of facilitating positive, responsible, healthier interpersonal relationships among early adolescents—as do these young people themselves.

## Future Directions

This study sought to bring about an understanding of the influence of participation in a program geared toward the positive development of adolescents. The *Lions Quest* program aims to prevent or delay adolescents’ alcohol and drug use by helping them to develop a constructive engagement in their school community through the development of social skills and competencies. The study offers distinctive insights from the perspective of those concerned by the implementation of the program in question. Nevertheless, it would be interesting to conduct a longitudinal study to assess the effect of *Lions Quest* over time.

As established earlier, there is little or no research calling for more studies that focus on the views of young adolescents. In fact, we propose that it is logical, and perhaps imperative, to consider the views of the very individuals for whom interventions are developed and implemented (Gallé and Lingard 2010). Moreover, our study indicates that they are well capable of reflecting, sharing their opinions, and offering ideas that are sometimes different from what it is expected of them, in studies that derive understanding through informal conversations.
